# Mechanistic insights into the health benefits of fish-oil supplementation against fine particulate matter air pollution: a randomized controlled trial

**DOI:** 10.1186/s12940-022-00908-1

**Published:** 2022-10-29

**Authors:** Lu Zhou, Yixuan Jiang, Zhijing Lin, Renjie Chen, Yue Niu, Haidong Kan

**Affiliations:** 1 School of Public Health, Key Lab of Public Health Safety of the Ministry of Education, NHC Key Lab of Health Technology Assessment, Fudan University, 200030 Shanghai, China; 2 Shanghai Key Laboratory of Meteorology and Health, Shanghai Typhoon Institute/CMA, 200030 Shanghai, China; 3 Department of Environmental Health, School of Public Health, Fudan University, 200032 Shanghai, China

**Keywords:** Fine particulate matter, Fish oil, Randomized controlled trial, Metabolomics

## Abstract

**Background:**

Dietary fish-oil supplementation might attenuate the associations between fine particulate matter (PM_2.5_) and subclinical biomarkers. However, the molecular mechanisms remain to be elucidated. This study aimed to explore the molecular mechanisms of fish-oil supplementation against the PM_2.5_-induced health effects.

**Methods:**

We conducted a randomized, double-blinded, and placebo-controlled trial among healthy college students in Shanghai, China, from September 2017 to January 2018. A total of 70 participants from the Fenglin campus of Fudan University were included. We randomly assigned participants to either supplementation of 2.5-gram fish oil (n = 35) or sunflower-seed oil (placebo) (n = 35) per day and conducted four rounds of health measurements in the last two months of the trial. As a *post hoc* exploratory study, the present untargeted metabolomics analysis used remaining blood samples collected in the previous trial and applied a Metabolome-Wide Association Study framework to compare the effects of PM_2.5_ on the metabolic profile between the sunflower-seed oil and fish oil groups.

**Results:**

A total of 65 participants completed the trial (34 of the fish oil group and 31 of the sunflower-seed oil group). On average, ambient PM_2.5_ concentration on the day of health measurements was 34.9 µg/m^3^ in the sunflower-seed oil group and 34.5 µg/m^3^ in the fish oil group, respectively. A total of 3833 metabolites were significantly associated with PM_2.5_ in the sunflower-seed oil group and 1757 in the fish oil group. Of these, 1752 metabolites showed significant between-group differences. The identified differential metabolites included arachidonic acid derivatives, omega-3 fatty acids, omega-6 fatty acids, and omega-9 fatty acids that were related to unsaturated fatty acid metabolism, which plays a role in the inflammatory responses.

**Conclusion:**

This trial suggests fish-oil supplementation could mitigate the PM_2.5_-induced inflammatory responses via modulating fatty acid metabolism, providing biological plausibility for the health benefits of fish-oil supplementation against PM_2.5_ exposure.

**Trial registration:**

This study is registered at ClinicalTrails.gov (NCT03255187).

**Supplementary Information:**

The online version contains supplementary material available at 10.1186/s12940-022-00908-1.

## Introduction

Fine particulate matter (PM_2.5_) has been a major air pollutant threatening human health. Epidemiological and experimental studies have closely associated PM_2.5_ pollution with a series of adverse health outcomes [1–3]. The Global Burden of Disease project estimated that ambient particulate matter pollution accounted for 4.14 million deaths worldwide in 2019 [2].

Although the PM_2.5_ concentration has decreased in China as a result of a series of clean air actions [4], PM_2.5_ levels are still above the guideline of the World Health Organization. In this context, effective personal protective measures are recommended. Previous investigations demonstrated that the applications of air purifiers and particulate-filtering respirators could significantly reduce the damage caused by PM_2.5_ exposure [5, 6]. However, these measures have limitations in some settings (e.g., outdoors and in rural areas), where dietary intervention might offer a simple and effective approach to protect individual health.

Fish oil is one of the common dietary supplements, with an abundance of long-chain omega-3 polyunsaturated fatty acids as the active ingredients, including eicosapentaenoic acid (EPA) and docosahexaenoic acid (DHA) [7, 8]. Although the protective effects of fish-oil supplementation on the prevention and treatment of diseases remain controversial, current evidence has supported its effectiveness in susceptible populations [9]. Previous studies found that dietary fish-oil supplementation could alleviate PM_2.5_-induced changes in biomarkers of systemic inflammation, coagulation, endothelial dysfunction, and oxidative stress [10–13]. However, these studies only considered a few pre-selected biomarkers of interest based on literature, and the exact mechanisms remain to be fully elucidated.

In the last few years, untargeted metabolomics has become a sensitive tool to comprehensively and directly explore metabolic perturbations associated with air pollution [14–16]. Compared to targeted technologies, it allows for unbiased detection and exploration of potential biological mechanisms and molecules, thus facilitating non-hypothesis driven research. Our previous study has reported that fish-oil supplementation could attenuate the associations between PM_2.5_ and a series of subclinical biomarkers in a randomized, double-blinded, and placebo-controlled trial in healthy young adults in China [10]. Accordingly, the present metabolomic analysis, as a *post hoc* exploratory study, used remaining blood samples collected in the previous trial to provide insights into the biological mechanisms for the potential health benefits of fish-oil supplementation against PM_2.5_ exposure.

## Methods

### Participants and study design

This trial was conducted from September 2017 to January 2018 in Shanghai, China. A total of 70 college students living and studing on the Fenglin campus of Fudan University were recruited. We included people who were healthy, aged ≥ 18 years, and residing on campus during the study period. None of the participants reported exposures to fumes of any kind (including cooking and second-hand tobacco smoke) during the study period as smoking or cooking is not allowed on this campus. We excluded those participants who currently or formerly smoked, who regularly consumed alcohol, who had allergic reactions to omega-3 fatty acids, or who were diagnosed cardiovascular, respiratory diseases or infectious diseases. Detailed inclusion and exclusion criteria have been described previously [10].

We randomly assigned all eligible participants to either the fish oil group or the sunflower-seed oil (placebo) group and asked them to take two capsules of marine-derived fish oil or sunflower-seed oil per day, respectively, throughout the study period (four months). Each capsule of fish oil was 1.25 g and contained 36.0% EPA and 24.0% DHA in total. Sunflower-seed oil capsules were identical in appearance and weight to the fish oil capsules, and contained 14.4% palmitic acid, 16.0% oleic acid, and 57.6% linoleic acid. Sunflower-seed oil was commonly used as placebo in previous trials of fish-oil supplementation [17, 18]. In the present trial, we did not find any significant health benefits on subclinical outcomes after sunflower-seed oil supplementation [10]. In the first two months of the trial, we asked all participants to receive either fish oil or sunflower-seed oil intervention to keep a relatively stable biologically effective dose of fish oil. In the latter two months of the trial, four rounds of follow-ups were arranged at the 8th, 10th, 12th, and 14th week after the start of fish-oil or sunflower-seed oil supplementation for each participant to assess the metabolic perturbations associated with ambient PM_2.5_ concentration (see Figure S1). For participants, their four follow-ups were scheduled on different days to expand the exposure variations. Therefore, the dates of measurements were distributed across the winter season (i.e., November, December, and January) of 2017–2018. For each participant, all his or her blood samples were collected on the same day of the week and at a fixed time (8:00 to 9:00 am) to avoid the influence of biological rhythm. All participants were required to complete food frequency questionnaires to record their dietary intake of nutrients during the study period. To evaluate their compliance to the intervention, we measured the circulating concentrations of DHA, EPA, and oleic acid at baseline and over follow-ups. Both participants and study staff were blinded to the group assignment.

The protocol of this study was approved by the Institutional Review Board at School of Public Health, Fudan University and registered at ClinicalTrails.gov (NCT03255187). Written informed consent was obtained from each participant.

### Exposure measurements

An Environmental Dust Monitor (GRIMM Aerosol Technik Ainring, Ainring, Germany) was installed on the rooftop of a 10-meter-high building in the center of the Fenglin campus, far from major sources of emission. Thus, its measurements may well reflect the general background levels of PM_2.5_ around the campus. The monitor was set to record the concentrations of ambient PM_2.5_ every minute, from which we calculated the hourly average PM_2.5_ concentrations only when > 75% of the records were available within this period. Then, we further calculated the PM_2.5_ concentrations at various average periods prior to the collection of blood samples (i.e., lags of 0–3 h, 0–6 h, 0–12 h, 0–24 h, and 0–48 h) when > 75% of the hourly data were available.

To control for the confounding effects of gaseous pollutants, we also collected the hourly concentrations of nitrogen dioxide (NO_2_), sulfur dioxide (SO_2_), ozone (O_3_), and carbon monoxide (CO) from the nearest fixed-site monitor to the campus (approximately 3.5 km). Besides, we used portable loggers (HOBO UX100-003, Onset Computer, Bourne, Massachusetts) to record temperature and relative humidity at the individual level.

### Sample collection and metabolomics analyses

The blood samples were immediately incubated at 37 °C for 15 min and then were centrifuged at low speed for 15 min to acquire serum. The collected serum samples were then split into several aliquots and stored at -80 °C within 30 min to minimize the potential changes in metabolites.

Before analysis, serum samples were prepared following a standard procedure. We conducted an untargeted metabolomics analysis by using ultrahigh performance liquid chromatography with high resolution mass spectrometry (UPLC-HRMS) techniques on VION IMS QTOF Mass Spectrometer coupled with the AQCQUITY UPLC I-Class system (Waters Corporation, Milford, US). Serum metabolome was analyzed in both positive and negative electrospray ionization modes. Serum samples were analyzed in random order within the same analytical run, and quality control samples were added into the sequence every 10 samples to evaluate the data repeatability. The mass spectral data were processed using Proqenesis QI software (Waters Corporation, Milford, US) to obtain a data matrix, including mass-to-charge ratio (*m/z*), retention time, ion intensity, and sample information. To ensure data quality, we excluded metabolic features detected in less than 50% of samples. All ion intensity data were log-transformed for normalization. More details of the sample preparation, UPLC-HRMS acquisition, quality control and data processing are described in the methods section of the supplementary material.

### Metabolome-wide association study (MWAS)

We followed the Metabolome-Wide Association Study workflow to compare the effects of PM_2.5_ on serum metabolome in the two groups [19, 20], and the flowchart of analyses is presented in the Figure S2 in the supplementary material. The MWAS approach allows for metabolic pathway analyses without prior knowledge of the features’ chemical identity.

First, we used linear mixed effect (LME) models to examine the associations between metabolic features’ intensity and ambient PM_2.5_ concentration in both groups and both ionization modes, respectively (a total of 4 sets of models). We set a random intercept for each participant in the LME model to account for within-participant correlations. We also adjusted the following covariates as in the previous study[10]: age, sex, body mass index (BMI), and natural cubic spline smooth functions of 24-hour average temperature and 24-hour average relative humidity using 3 degrees of freedom for both. The feature with a *P*-value < 0.05 was considered to be significantly associated with ambient PM_2.5_ concentration.

Afterwards, we applied Z-tests to examine the between-group differences in the estimated effects of PM_2.5_ on metabolic features:

$${\rm{Z}} = \frac{{(\beta 1 - \beta 2)}}{{\sqrt {{\rm{S}}{{\rm{e}}_1}^2 + {\rm{S}}{{\rm{e}}_2}^2} }}$$

where β1 and β2 were the effect estimates of PM_2.5_ on a certain metabolite in the fish oil group and the sunflower-seed oil group, respectively; Se_1_ and Se_2_ were their respective standard errors; Z was the Z-test score for pairwise comparison.

We defined differential metabolites as those that were significantly associated with ambient PM_2.5_ concentration in at least one group and showed significant between-group differences associated with PM_2.5_. We annotated them by searching the Human Metabolome Database (HMDB), METLIN (http://metlin.scripps.edu/index.php), and LIPID MAPS (https://www.lipidmaps.org/) with a mass error threshold of 10 ppm. Furthermore, we used tandem mass spectrometry (MS/MS) to fragment annotated metabolites and confirm the identification by comparing MS/MS spectra to authentic chemical references when available in the public libraries. For the confirmed metabolites, we applied an interaction analysis by including the interaction term (“PM_2.5_ concentration × treatment”) in the LME models. The interactive effect was considered statistically significant when the *P*-value of interaction term less than 0.05.

Additionally, we performed metabolic pathway analyses for differential metabolites in each ionization mode, separately, using mummichog (Version 1.0.10). Mummichog is a novel biostatistical application that infers biological pathways directly from spectral feature output without prior metabolite annotation [21]. To minimize false positive discovery, we restricted the adduct forms to the following: M^[1+]^, M + H^[1+]^, M + Na^[1+]^ for the positive ionization mode; M-H^[−]^, M-2 H^[2−]^ and M-H_2_O-H^[−]^,for the negative ionization mode. The pathway with an adjusted *P*-value < 0.05 was considered to be statistically significant.

Finally, we conducted two sensitivity analyses to test the robustness of our results. First, we adjusted for the intake of dietary nutrients that differed significantly between groups over follow-ups. Second, we fitted two-pollutant models by separately adjusting for the concentrations of gaseous pollutants (i.e., NO_2_, SO_2_, O_3_, and CO) at lag 0–24 h.

The LME models were fitted in R software (Version 4.0.3, R Project for Statistical Computing) using the ‘*lme4*’ package. The associations between PM_2.5_ and metabolites were expressed as percentage changes in metabolites and their 95% confidence intervals (CIs) per 10-µg/m^3^ increase in ambient PM_2.5_ concentrations. The pathway analyses were performed in the mummichog server (mummichog-2.appspot.com).

## Results

### Descriptive statistics

In total, 65 participants completed the scheduled following-ups (34 of the fish oil and 31 of the sunflower-seed oil group), and five participants withdrew from the trial. As shown in Table 1, the mean age of the participants in the sunflower-seed oil group and fish oil group was 22.9 years (SD = 1.3 years) and 23.0 years (SD = 2.3 years), respectively, while the mean BMI was 21.6 kg/m^2^ (SD = 3.0 kg/m^2^) and 21.7 kg/m^2^ (SD = 3.2 kg/m^2^), respectively. There was no significant difference in the characteristics of participants (age, sex and BMI) between groups at baseline. Our previous publication has also reported good comparability of baseline physiological conditions between groups [10]. Besides, the intake of dietary nutrients was similar except for heptadecanoic acid and heptadecenoic acid. Compliance to the intervention has also been reported previously that the participants in the fish oil group had obviously higher levels of EPA and DHA over follow-ups compared to their baseline levels and participants in the sunflower-seed oil group [10].

The proportion of missing data was less than 5% for the minute-level concentrations of PM_2.5_, which occurred randomly. There were no missing data at hourly level. As presented in Table 1, the mean concentrations of PM_2.5_ on the day of health measurements in the sunflower-seed oil and the fish oil groups were 34.9 µg/m^3^ (SD = 16.1 µg/m^3^) and 34.5 µg/m^3^ (SD = 15.5 µg/m^3^), respectively. The concentrations of PM_2.5_ and gaseous air pollutants were comparable between the two groups. The mean levels of temperature were 19.6 °C (SD = 1.4 °C) and 19.0 °C (SD = 2.1 °C) while the mean humidity was 42.4% (SD = 8.2%) and 47.0% (SD = 12.6%) in the sunflower-seed oil and fish oil groups, respectively.

**Table 1:** Summary statistics on individual characteristics, air pollutants and personal meteorological factors during 0–24 h before blood sample collection

Variables	Sunflower-seed oil group (*n* = 31)	Fish oil group (*n* = 34)	*P*-value*
Age, years, mean (SD)	22.9 (1.3)	23.0 (2.3)	0.73
Female, *n* (%)	18 (58%)	20 (59%)	0.95
BMI, kg/m^2^, mean (SD)	21.6 (3.0)	21.7 (3.2)	0.84
PM_2.5_, µg/m^3^, mean (SD)	34.9 (16.0)	34.5 (15.5)	0.83
NO_2_, µg/m^3^, mean (SD)	58.3 (29.9)	53.9 (20.4)	0.20
SO_2_, µg/m^3^, mean (SD)	12.5 (5.2)	12.6 (3.8)	0.96
O_3_, µg/m^3^, mean (SD)	38.9 (19.5)	37.9 (14.7)	0.65
CO, mg/m^3^, mean (SD)	0.9 (0.3)	1.0 (0.2)	0.59
Temperature, °C, mean (SD)	19.6 (1.4)	19.0 (2.1)	0.02
Relative humidity, %, mean (SD)	42.4 (8.2)	47.0 (12.6)	0.01

**Abbreviations**: SD, standard deviation; PM_2.5_, particulate matter with aerodynamic diameter ≤ 2.5 μm; NO_2_, nitrogen dioxide; SO_2_, sulfur dioxide; O_3_, ozone; CO, carbon monoxide

***Note.*** *A t-test or chi-square test was used to compare the differences between the sunflower-seed oil and the fish oil groups

### Identified metabolites and pathways

**Fig. 1:**
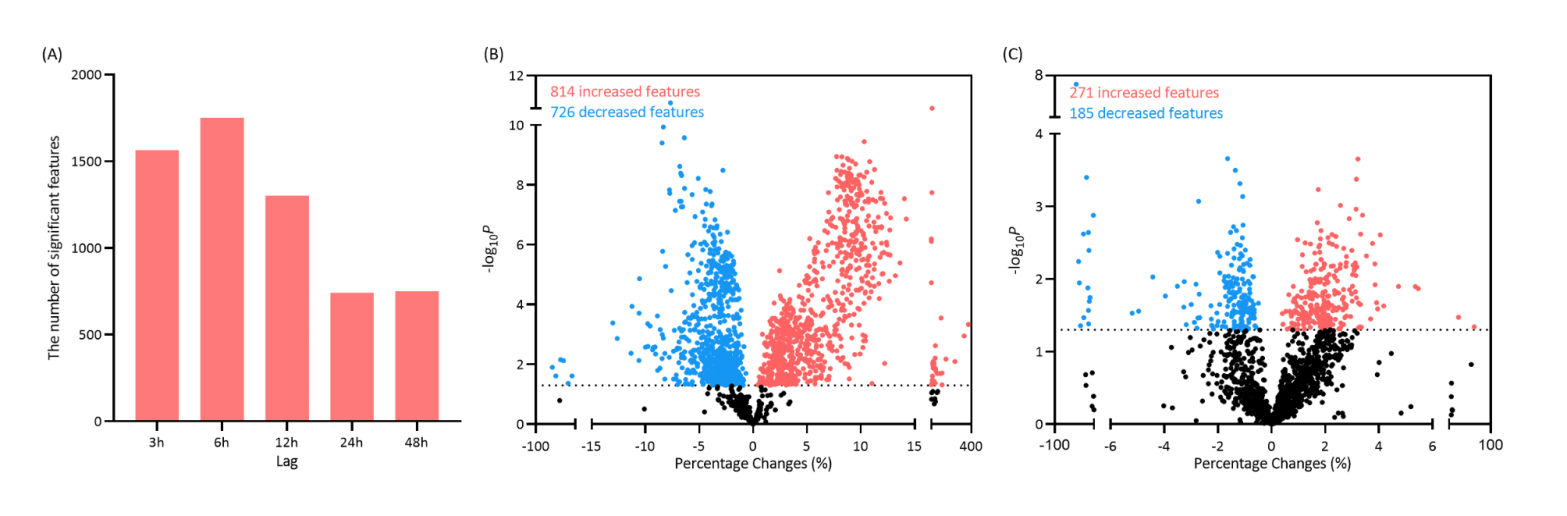
Summary description of differential metabolic features. (A) The number of features showing significant between-group differences associated with ambient fine particulate matter (PM_2.5_) concentration in the sunflower-seed oil (placebo) and the fish oil groups at different lag periods. Volcano plots of percentage changes associated with a 10-µg/m^3^ increment in ambient PM_2.5_ concentration versus –log_10_*P* for differential metabolites in the sunflower-seed oil group (B) and the fish oil group (C) at a lag of 0–6 h

The numbers of differential metabolites between the two groups varied by the lags of exposure. As shown in Fig. 1 A, the sum of differential metabolites increased within 6 h after exposure and then presented a dramatic decline. Therefore, we chose the lag of 0–6 h for the main analyses. At a lag of 0–6 h, a total of 3833 metabolites were significantly associated with PM_2.5_ in the sunflower-seed oil group, 1757 in the fish oil group, and 4751 in at least one group. Among these 4751 metabolites, 1752 metabolites showed significant between-group differences. Overall, most differential metabolites were significantly associated with ambient PM_2.5_ concentration in the sunflower-seed oil group, while the associations became non-significant and weaker in the fish oil group. According to the LME models, a total of 1540 differential metabolites changed significantly after PM_2.5_ exposure at a condition of *P*-value < 0.05 in the sunflower-seed oil group and 456 in the fish oil group (see Fig. 1 B and C).

**Fig. 2:**
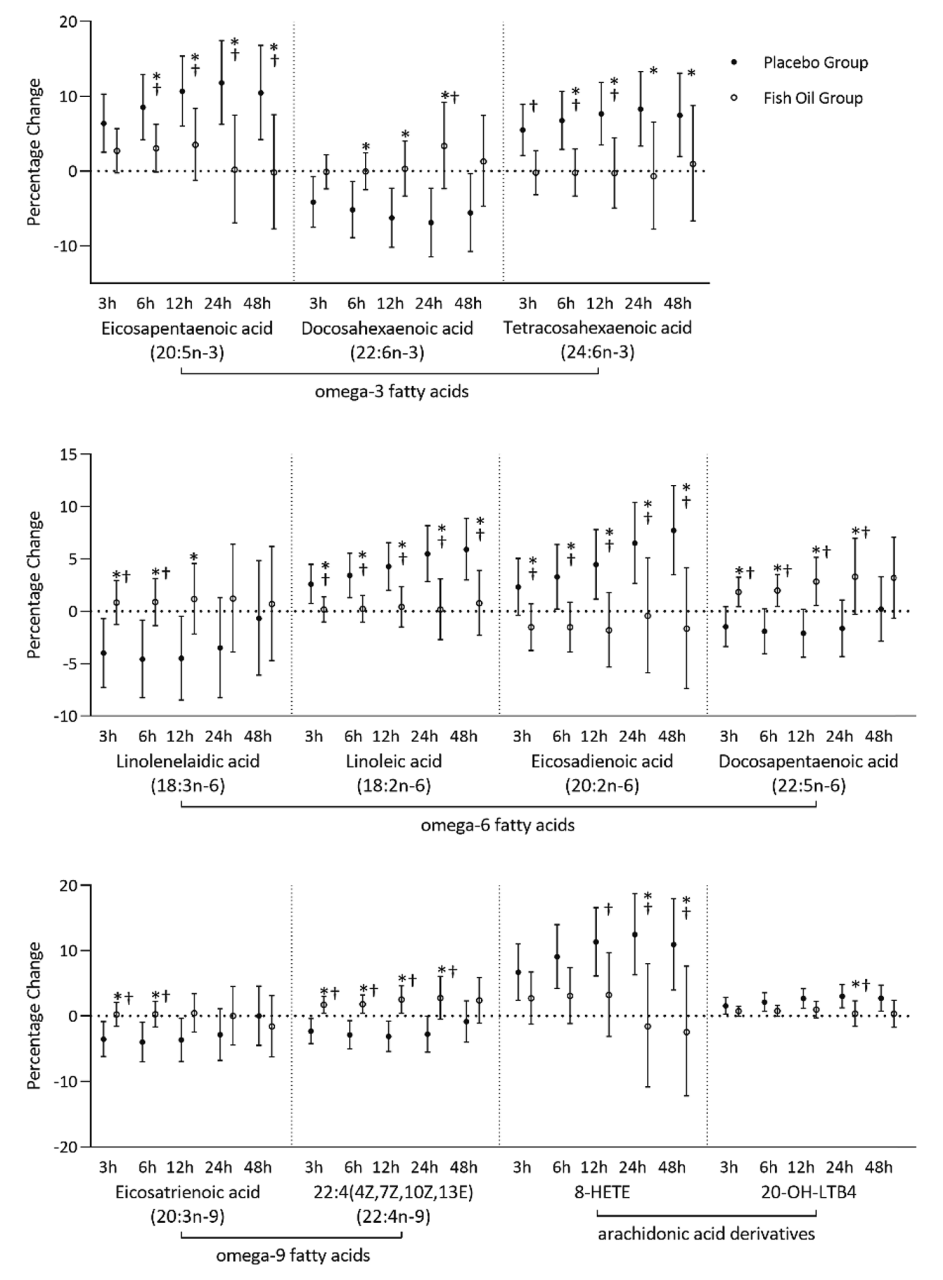
Percentage changes in omega-3 fatty acids, omega-6 fatty acids, omega-9 fatty acids, and arachidonic acid derivatives associated with a 10-µg/m^3^ increment in ambient fine particulate matter (PM_2.5_) concentration in the sunflower-seed oil (placebo) group and the fish oil group. **Abbreviations**: 8-HETE, 8-hydroxyeicosatetraenoic acid; 20-OH-LTB4, 20-OH-Leukotriene B4. Note.* Significant difference between groups (*P*-value < 0.05). † Significant interaction between PM_2.5_ concentration and treatment (*P*-value < 0.05)

In total, 71 differential metabolites were identified. Among them, 33 and 38 metabolites were from the positive ionization mode and negative ionization mode, respectively. We further confirmed the identification of 11 features by matching MS/MS spectral. Figure 2 showed the percentage changes in the confirmed metabolites belonging to polyunsaturated fatty acids and their derivates, including arachidonic acid derivatives, omega-3 fatty acids, omega-6 fatty acids, and omega-9 fatty acids. The direction and magnitudes of these changes varied substantially between groups.

As shown in Fig. 2, the metabolites of arachidonic acid, 8-hydroxyeicosatetraenoic acid (8-HETE) and 20-Hydroxy-Leukotriene B4 (20-OH-LTB4), were significantly associated with ambient PM_2.5_ concentration in the sunflower-seed oil group, but not in the fish oil group. At a lag of 0–24 h, significant between-group differences were observed for both metabolites, and the effects of PM_2.5_ exposure were the strongest in the sunflower-seed oil group. Each 10-µg/m^3^ increase in ambient PM_2.5_ concentration was associated with increases of 12.34% (95%CI: 6.28%, 18.75%) in 8-HETE and 3.00% (95%CI: 1.24%, 4.80%) in 20-OH-LTB4 in the sunflower-seed oil group. The corresponding changes were − 1.87% (95%CI: -10.81%, 7.97%) and 0.36% (95%CI: -1.57%, 2.32%) in the fish oil group. Besides, we observed significant between-group differences for other polyunsaturated fatty acids at a lag of 0–6 h (see Fig. 2). For instance, each 10-µg/m^3^ increment in ambient PM_2.5_ concentration was associated with changes of -5.24% (95%CI: -8.93%, -1.40%) in DHA (22:6n-3), 3.40% (95%CI: 1.30%, 5.55%) in linoleic acid (18:2n-6), and − 4.00% (95%CI: -6.98%, -0.94%) in eicosatrienoic acid (20:3n-9) in the sunflower-seed oil group. The corresponding changes were − 0.06% (95%CI: -2.49%, 2.43%), 0.24% (95%CI: -1.03%, 1.53%), and 0.26% (95%CI: -1.68%, 2.24%) in the fish oil group. In addition, we observed statistically significant interactions between PM_2.5_ concentration and treatment, with greater associations between PM_2.5_ concentration and metabolites in the sunflower-seed oil group than in the fish oil group, suggesting a possible effect modification by fish-oil supplementation on PM_2.5_-induced metabolic perturbations (see Fig. 2).

Table S3–S4 summarize 60 identified but not confirmed metabolites associated with PM_2.5_ in positive and negative ionization modes. We presented their percentage changes associated with each 10-µg/m^3^ increase in ambient PM_2.5_ concentration at a lag of 0–6 h. Generally, most of these metabolites were glycerophospholipids, including phosphatidyl choline (PC), phosphatidyl inositol (PI), phosphatidic acid (PA), lysophosphatidyl choline (LysoPC), phosphatidyl serine (PS), and phosphatidyl ethanolamine (PE). For example, we found a 10-µg/m^3^ increment in ambient PM_2.5_ concentration was associated with an increase of 2.69% (95%CI: 0.80%, 4.61%) in sphingosine-1-phosphate (S1P) in the sunflower-seed oil group. However, the association became negative and lost significance in the fish oil group (see Table S3).

**Fig. 3:**
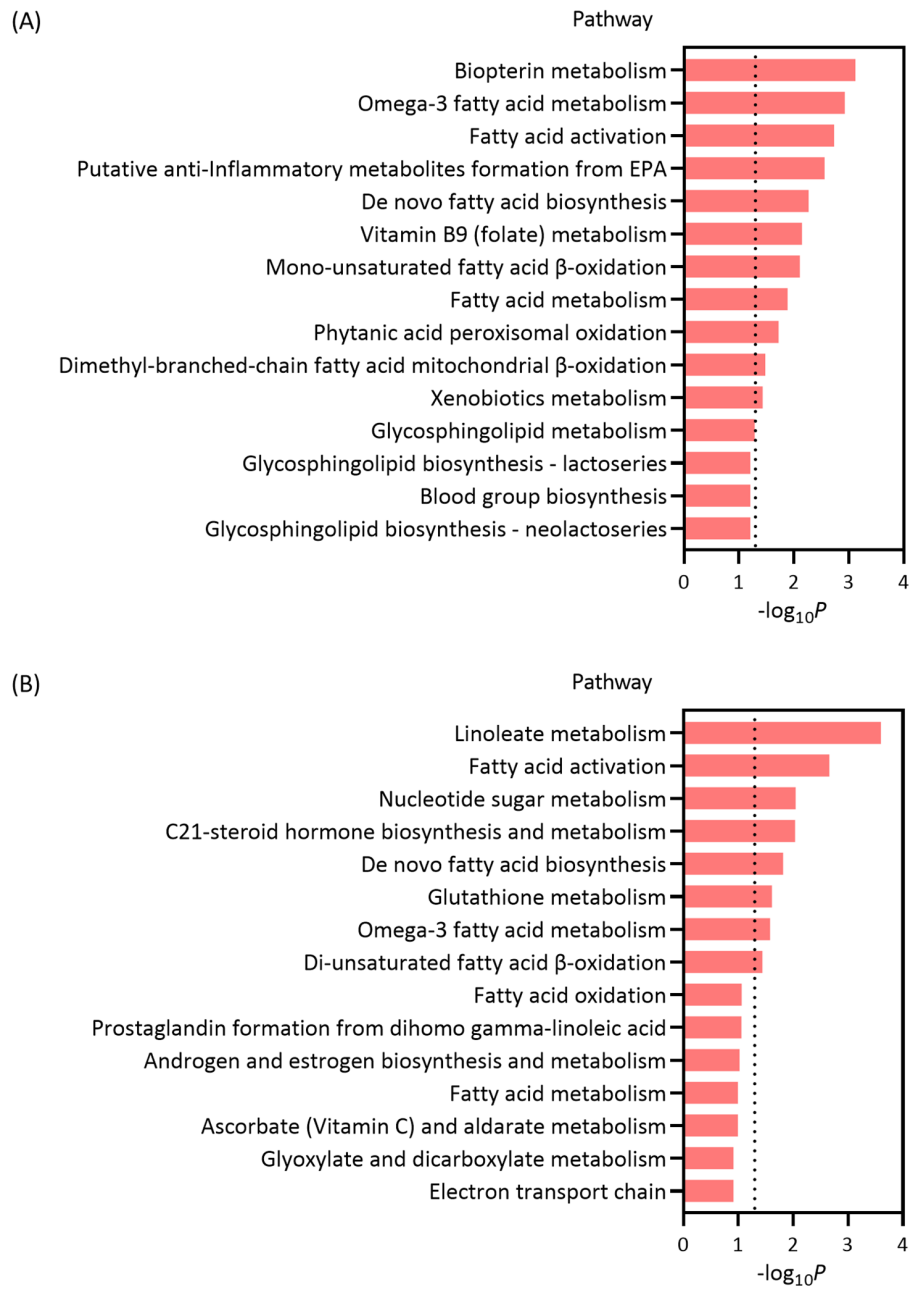
Top 15 most enriched metabolic pathways in positive ionization mode (A), and negative ionization mode (B) at a lag of 0–6 h

Figure 3 illustrates the top 15 most enriched pathways, of which 11 and 8 were significantly enriched in positive and negative ionization mode, respectively. The pathways were mainly enriched in the processes of fatty acid metabolism, especially polyunsaturated fatty acids metabolism, including omega-3 fatty acid metabolism, fatty acid activation, putative anti-inflammatory metabolites formation from EPA, de novo fatty biosynthesis, fatty acid β-oxidation, fatty acid metabolism, and linoleate metabolism.

In the sensitivity analyses, after adjusting for dietary intake of nutrients, the results of 11 confirmed metabolites were almost unchanged (see Figure S3), and the top pathways remained significant (see Figure S4). In addition, these findings were not substantially changed after adjusting for four gaseous air pollutants in two-pollutant models (see Figure S5–S8).

## Discussion

We conducted a randomized placebo-controlled trial and applied an untargeted serum metabolomics approach to explore the potential biological mechanism by which fish-oil supplementation could alleviate the PM_2.5_-induced health effects. In this study, we found that ambient PM_2.5_ concentration was associated with several metabolites related to polyunsaturated fatty acid metabolism and inflammation in the sunflower-seed oil (placebo) group rather than in the fish oil group. We also found fish-oil supplementation could mitigate or even inverse the effects of PM_2.5_ exposure on pro-inflammatory metabolites. Our findings add biological plausibility of the health benefits of fish-oil supplementation against PM_2.5_ exposure from a mechanistic perspective.

In our study, the metabolites that showed significant between-group differences in association with PM_2.5_ were mainly polyunsaturated fatty acids and their derivates, some of which were involved in the biosynthesis and metabolism of arachidonic acid. Arachidonic acid metabolism is a potent regulator of inflammation [22]. We found that ambient PM_2.5_ concentration was positively associated with 8-HETE and 20-OH-LTB4, the products of arachidonic acid metabolism, in the sunflower-seed oil group. These metabolites were lipid mediators of an inflammatory process and might be related to a series of cardiovascular diseases[23, 24]. However, such associations became much weaker and lost significance in the fish oil group, indicating that fish-oil supplementation may have the potential to inhibit PM_2.5_-related inflammatory reactions by affecting arachidonic acid metabolism. Results from our previous study supported these findings that fish-oil supplementation could attenuate the inflammatory effect of PM_2.5_ [10, 13] and further play a protective role against the development and progression of chronic inflammatory-related diseases [7, 25].

In addition to arachidonic acid derivates, we observed between-group differences in the effects of PM_2.5_ among a series of polyunsaturated fatty acids, including omega-3, omega-6, and omega-9 fatty acids. Among omega-3 fatty acids, DHA was negatively associated with ambient PM_2.5_ concentration in the sunflower-seed oil group. As previous studies reported, DHA have the potential to produce resolvins and maresins to treat acute inflammatory responses [26]. However, we did not find a significant reduction in DHA in the fish oil group. One possible speculation is that DHA from the fish oil might compensate for the loss of DHA induced by PM_2.5_. Besides, linoleic acid was found to be positively associated with PM_2.5_ in the sunflower-seed oil group. Linoleic acid, a predominant omega-6 fatty acid, could be metabolized to arachidonic acid and subsequently produced large amounts of pro-inflammatory mediators. Our previous findings in arachidonic acid derivates also supported the elevation of linoleic in the sunflower-seed oil group. For omega-9 fatty acids, we observed PM_2.5_ led to a significant decrease in eicosatrienoic acid in the sunflower-seed oil group. Eicosatrienoic acid (20:3n-9), also called mead acid, is de novo synthesized by humans. It is pretty stable and has been reported to protect health by anti-lipid peroxidation. Meanwhile, its metabolite, Leukotriene A3, was also reported to serve as an anti-inflammatory mediator by inhibiting the synthesis of Leukotriene B4 [27]. Almost unchanged eicosatrienoic acid (20:3n-9) in the fish oil group suggested that fish oil may alleviate PM_2.5_-induced inflammatory responses or oxidative stress through modulating omega-9 fatty acid metabolism.

It should be mentioned that fish oil consumption could inverse the associations between PM_2.5_ and some metabolites with anti-inflammatory potential like DHA and mead acid, although these inverse associations were non-significant. The possible explanation might be that fish oil may alleviate PM_2.5_-induced inflammation responses by enhancing anti-inflammatory capacity. Consistently, previous studies revealed the anti-inflammatory and pro-resolving role of fish oil in the course of acute inflammation [22]. Researches also showed that fish-oil supplementation could increase the circulating levels of anti-inflammatory biomarkers [28, 29].

Moreover, we found a considerable number of phospholipids in relation to ambient PM_2.5_ concentration. Generally, the magnitudes of changes were much larger in the sunflower-seed oil group when compared with those in the fish oil group. Meanwhile, most of the associations inversed and/or lost significance in the fish oil group. Phospholipids comprise a large number of bioactive lipids that have widespread effects on physiological processes. Notably, their complex metabolism networks and signaling systems are pivotal in regulating innate immunity and maintaining homeostasis [30]. Through dynamic changes, phospholipids and their derivates play complex roles in multiple activities related to various diseases [30]. For instance, we found S1P, a signaling lipid, was positively associated with PM_2.5_ in the sunflower-seed oil group. S1P was reported to promote cardiovascular injury in physiological and pathological processes, resulting in acute cardiac responses and chronic cardiovascular diseases [31]. However, the association became non-significant in the fish oil group, indicating the potential health benefits of fish oil supplementation.

In the pathway analyses, differential metabolites were mainly enriched in 19 biological pathways. Most of them were involved in fatty acid biosynthesis and metabolism, including omega-3 fatty acid metabolism, fatty acid activation, de novo fatty acid biosynthesis, fatty acid metabolism, and linoleate metabolism. These results further supported our main findings that fish-oil supplementation might reduce PM_2.5_-induced fluctuations in fatty acid metabolism. Consistently, previous study also indicated health benefits of fish-oil supplementation against adverse lipid effects [12]. Perturbations in fatty acid metabolism have been found in most studies [14, 32] as potential mediators linking PM_2.5_ pollution to adverse cardiovascular effects. Accordingly, we speculated that fish-oil supplementation might reduce the cardiovascular damage of PM_2.5_ by modulating fatty acid metabolism.

Collectively, we found fish oil had health benefits against PM_2.5_ exposure mainly through modulating unsaturated fatty acid metabolism, which plays a role in the inflammatory response. It is biologically plausible. A pharmacology research manifested that the main components of fish oil, EPA and DHA, could prevent the formation of eicosanoids from arachidonic acid via substrate competition [22]. Alternative eicosanoids derived from EPA or DHA were proved to have less pro-inflammatory actions than the eicosanoids derived from arachidonic acid, and some even had anti-inflammatory actions [26]. Therefore, fish-oil supplementation could alter the balance of eicosanoid production towards a less inflammatory profile [22].

Our study still has several limitations. First, our sample size was relatively small, which might limit the ability to detect a significant difference in metabolites between the two groups perturbated by PM_2.5_ exposure. Second, exposure measurement errors were possible because exposure data were obtained from a fixed-site monitor within the campus (for PM_2.5_) and a nearby fixed-site outside the campus (for gaseous air pollutants). However, we believe these non-differential errors between groups would not substantially change our results. Third, we could not fully exclude the confounding effects of dietary nutrients intake. However, we believe this did not have substantial effects on our results because all participants had a regular diet at the campus cafeteria and our results were barely changed after adjusting for the roughly estimated dietary intake. Finally, due to budget constraints, we did not measure baseline serum metabolome and we cannot fully rule out the potential effects of sunflower-seed oil supplementation on metabolome.

## Conclusion

This randomized placebo-controlled trial suggests that fish-oil supplementation could mitigate the inflammatory responses induced by PM_2.5_ exposure via modulating fatty acid metabolism. Our findings provide a biological mechanistic insight into the health benefits of fish-oil supplementation against PM_2.5_ air pollution.

## Electronic supplementary material

Below is the link to the electronic supplementary material.

Supplementary Material 1

## Data Availability

The datasets used and/or analyzed during the current study are available from the corresponding authors on reasonable request.

## Abbreviations

PM_2.5_: Fine particulate matter
EPA: Eicosapentaenoic acid
DHA: Docosahexaenoic acid
UPLC-HRMS: Ultrahigh performance liquid chromatography with high-resolution mass spectrometry
LME: Linear mixed effect
BMI: Body mass index
MS/MS: Tandem mass spectrometry
CI: Confidence interval
8-HETE: 8-hydroxyeicosatetraenoic acid
20-OH-LTB4: 20-Hydroxy-Leukotriene B4
PC: Phosphatidyl choline
PI: Phosphatidyl inositol
PA: Phosphatidic acid
LysoPC: Lysophosphatidyl choline
PS: Phosphatidyl serine
PE: Phosphatidyl ethanolamine
S1P: Sphingosine-1-phosphate
