# Supplementary material for: Mechanistic insights into the health benefits of fish-oil supplementation against fine particulate matter air pollution: a randomized controlled trial
Source: Environ Health. 2022 Oct 29;21:104. doi: 10.1186/s12940-022-00908-1 (PMC9617415; doi:10.1186/s12940-022-00908-1)
Supplement: Supplementary file 1 — Supplementary Material 1 [file 12940_2022_908_MOESM1_ESM.docx]

**Supplementary Material**

**Methods**

**Sample Preparation**

Serum samples were prepared for untargeted metabolomics analysis according to the following procedures:

1. Samples stored at -80°C were thawed at room temperature. 10 μL of 2-chloro-l-phenylalanine (0.3 mg/mL) and 10 μL of 1-heptadecanoyl-2-hydroxy-sn-glycero-3-phosphocholine (0.01 mg/mL), both dissolved in methanol, were added to 100 μL of sample as internal standards, and the mixture was vortexed for 10 seconds.

2. 300 μL of a mixture of methanol and acetonitrile (V:V = 2:1) was added. The mixture was vortexed for 1 minutes, ultrasonicated in ice water for 10 minutes, and stored at -20°C for 30 minutes.

3. The mixture was centrifuged at 13000 rpm for 10 minutes at 4°C to obtain the supernatant. 300 μL of supernatant was dried in a freeze concentration centrifugal dryer. Next, 400 μL of a mixture of methanol and water (V:V = 1:4) was added, and then vortexed for 30 seconds and ultrasonicated for 2 minutes.

4. The mixture was centrifuged again at 13000 rpm for 10 min at 4°C to obtain the supernatant. 150 μL of the supernatant was collected using a crystal syringe, filtered through a 0.22-μm microfilter and transferred to ultrahigh performance liquid chromatography (UPLC) vials. The vials were stored at -80°C until ultrahigh performance liquid chromatography with high resolution mass spectrometry (UPLC-HRMS) analysis.

5. Aliquots of all samples were pooled to prepare quality control samples (QCs). Consistently, 2-chloro-l-phenylalanine (0.3 mg/mL) and 1-heptadecanoyl-2-hydroxy-sn-glycero-3-phosphocholine dissolved in the methanol were also added as internal standards.

**UPLC-HRMS Analysis**

ACQUITY UPLC I-Class system (Waters Corporation, Milford, US) coupled with VION IMS QTOF Mass spectrometer (Waters Corporation, Milford, US) was used to analyze the metabolic profiling in both positive and negative electrospray ionization modes. ACQUITY UPLC BEH C18 column (1.7 μm, 2.1 × 100 mm) was employed in both ionization modes. Water and a mixture of acetonitrile and methanol (V:V = 2:3), both containing 0.1% formic acid, were used as mobile phases A and B, respectively. Linear gradient is shown in table S1. The flow rate was 0.4 mL/min and the column temperature was 45°C. All samples were kept at 4 °C during the analysis. The injection volume was 1 μL.

**Table S1.** Linear gradient of C18 column

| **Time (min)** | **Phase A (%)** | **Phase B (%)** |
| --- | --- | --- |
| 0 | 99 | 1 |
| 1 | 70 | 30 |
| 2.5 | 40 | 60 |
| 6.5 | 10 | 90 |
| 8.5 | 0 | 100 |
| 10.7 | 0 | 100 |
| 10.8 | 99 | 1 |
| 13 | 99 | 1 |

Data acquisition was performed in full scan mode, with mass-to-charge ratios (*m/z*) ranging from 50 to 1000. Both positive and negative ionization modes were conducted following the same parameters of mass spectrometry as shown in table S2.

**Table S2.** Parameters of mass spectrometry

| **Parameter** | **Value** |
| --- | --- |
| Capillary voltage | 2.5 kV |
| Cone voltage | 40 V |
| Collision energies | 4 eV |
| Source temperature | 115 °C |
| Desolvation temperature | 450 m |
| Desolvation gas flow | 900 L/h |
| Mass range | 50–1000 amu |
| Scan time | 0.2 s |
| Interscan delay | 0.02 s |

Argon (99.999%) was used as collision-induced dissociation gas and nitrogen (> 99.5%) was employed as desolvation and cone gas. For lock mass correction, a 250 ng/mL standard solution of leucine-enkephalin in acetonitrile/water/formic acid (V:V:V = 50:49.9:0.1) was continuously infused (5 μL/min) through the reference probe and scanned every 30 seconds.

The QCs were injected every 10 samples throughout the analytical run to verify the data repeatability.

**Data Processing**

The mass spectral data were processed using Progenesis QI (Waters, Milford, MA, USA). After peak picking and alignment, data was normalized using the total intensity of peaks that are present in all samples under study as the normalization factor. Progenesis QI could search ID of compounds using up to five different criteria including exact mass, MS/MS fragments, isotope similarity, retention time and collisional cross-section by searching Human Metabolome Database (HMDB), METLIN (http://metlin.scripps.edu/index.php), and LIPID MAPS (https://www.lipidmaps.org/). Each metabolite was identified according to its ID score that was evaluated from the five ID criteria mentioned above. Endogenous metabolites with a score value > 30 were selected and identified. The full score is 60, and the score value of the compound is the higher the better in identification.

**Table S3.** Metabolic features with significant between-group differences in association with ambient fine particulate matter (PM_2.5_) concentration in the sunflower-seed oil (placebo) group and the fish oil group in positive ionization mode at a lag of 0–6 hours.

| ***m/z*** | **RT (min)** | **Chemical identity** | **Adduct form** | **Percentage changes and their 95% CIs** | |
| --- | --- | --- | --- | --- | --- |
|  |  |  |  | **Fish oil group** | **Placebo group** |
| 175.1074 | 4.61 | N-Acetylornithine | M+H[1+] | -1.10 (-2.83, 0.67) | -3.55 (-5.13, -1.94) |
| 203.0481 | 2.74 | D-Galactose | M+Na[1+] | 0.06 (-1.36, 1.49) | -4.55 (-6.52, -2.54) |
| 238.0837 | 4.70 | 3,4,5-Trimethoxycinnamic acid | M+H-H2O[1+] | 1.26 (-0.87, 3.44) | -1.21 (-2.33, -0.08) |
| 260.1853 | 4.59 | O-hexanoyl-R-carnitine | M+H[1+] | -0.47 (-2.47, 1.57) | -6.21 (-9.72, -2.57) |
| 264.2684 | 7.02 | Elaidamide | M+H-H2O[1+] | -0.24 (-1.64, 1.18) | 2.29 (0.27, 4.36) |
| 279.2316 | 8.39 | 13-hydroxyoctadecadienoic acid | M+H-H2O[1+] | 1.41 (0.12, 2.71) | -1.89 (-4.05, 0.31) |
| 285.1471 | 7.38 | Leukotriene F4 | M+2H[2+] | -0.76 (-1.98, 0.46) | 2.05 (0.06, 4.07) |
| 288.2166 | 5.38 | L-Octanoylcarnitine | M+H[1+] | 0.06 (-2.46, 2.65) | -7.04 (-11.28, -2.60) |
| 316.2479 | 5.95 | Decanoylcarnitine | M+H[1+] | 0.02 (-2.58, 2.68) | -6.67 (-10.91, -2.23) |
| 370.2953 | 6.62 | cis-5-Tetradecenoylcarnitine | M+H[1+] | 0.65 (-0.80, 2.13) | -4.45 (-7.10, -1.73) |
| 379.2491 | 7.02 | Sphingosine-1-phosphate | M+H-H2O[1+] | -0.41 (-1.72, 0.92) | 2.69 (0.80, 4.61) |
| 380.1827 | 7.44 | PC(4:0/4:0) | M+H-H2O[1+] | 0.74 (-0.64, 2.13) | 3.51 (1.77, 5.27) |
| 395.2200 | 9.02 | PA(15:1(9Z)/0:0) | M+H[1+] | 1.79 (0.61, 2.98) | -0.56 (-1.98, 0.88) |
| 395.7920 | 4.62 | PC(P-16:0/22:6(4Z,7Z,10Z,13Z,16Z,19Z)) | M+2H[2+] | -0.27 (-13.00, 14.32) | 31.68 (7.32, 61.56) |
| 396.3100 | 6.74 | N-stearoyl glutamic acid | M+H-H2O[1+] | 1.14 (-0.46, 2.77) | -3.85 (-6.71, -0.90) |
| 413.2804 | 7.50 | PI(21:0/12:0) | M+2H[2+] | -0.68 (-1.18, -0.18) | -1.69 (-2.38, -0.98) |
| 441.3285 | 7.47 | PI(O-20:0/18:0) | M+2H[2+] | -0.61 (-1.24, 0.03) | -1.87 (-2.71, -1.03) |
| 455.3446 | 7.94 | PI(O-20:0/20:0) | M+2H[2+] | -0.34 (-0.76, 0.09) | -1.1 (-1.73, -0.47) |
| 519.3325 | 7.44 | LysoPC(18:2(9Z,12Z)) | M+H-H2O[1+] | 0.39 (-0.91, 1.70) | 3.91 (1.80, 6.07) |
| 543.3323 | 7.42 | LysoPC(20:4(5Z,8Z,11Z,14Z)) | M+H[1+] | 0.10 (-1.49, 1.71) | 2.90 (1.00, 4.83) |
| 544.3424 | 6.39 | PC(20:4(5Z,8Z,11Z,14Z)/0:0) | M+H[1+] | -0.12 (-1.07, 0.83) | 1.78 (0.39, 3.18) |
| 546.3554 | 8.70 | LysoPC(20:3(5Z,8Z,11Z)) | M+H[1+] | 3.24 (0.08, 6.51) | -4.09 (-8.15, 0.16) |
| 566.3245 | 7.33 | LysoPC(20:4(8Z,11Z,14Z,17Z)) | M+Na[1+] | -0.16 (-1.49, 1.19) | 2.21 (0.34, 4.11) |
| 580.4007 | 8.92 | PC(16:0/5:0) | M+H[1+] | 1.60 (0.19, 3.04) | -1.43 (-3.37, 0.54) |
| 682.5562 | 10.67 | DG(19:1(9Z)/22:5(7Z,10Z,13Z,16Z,19Z)/0:0)[iso2] | M+H[1+] | 1.48 (0.21, 2.77) | -0.87 (-2.63, 0.92) |
| 759.5771 | 11.57 | PC(18:1(11Z)/16:0) | M+H-H2O[1+] | -0.37 (-1.22, 0.49) | 0.93 (0.05, 1.82) |
| 764.5577 | 9.06 | PC(16:0/20:4(5Z,8Z,11Z,14Z)) | M+H-H2O[1+] | 1.46 (0.36, 2.56) | -2.13 (-4.09, -0.13) |
| 850.6668 | 8.65 | PC(18:3(9Z,12Z,15Z)/24:0) | M+H-H2O[1+] | 3.53 (1.11, 5.99) | -0.27 (-2.83, 2.36) |
| 958.8210 | 5.67 | PC(24:0/24:0) | M+H[1+] | 1.77 (-2.05, 5.74) | 10.65 (5.90, 15.63) |

**Abbreviation:** CIs, confidence intervals; PC, phosphatidyl choline; PA, phosphatidic acid; PI, phosphatidyl inositol; LysoPC, lysophosphatidyl choline; DG, Diacylglycerol.

**Table S4.** Metabolic features with significant between-group differences in association with ambient fine particulate matter (PM_2.5_) concentration in the sunflower-seed oil (placebo) group and the fish oil group in negative ionization mode at a lag of 0–6 hours.

| ***m/z*** | **RT (min)** | **Chemical identity** | **Adduct form** | **Percentage changes and their 95% CIs** | |
| --- | --- | --- | --- | --- | --- |
|  |  |  |  | **Fish oil group** | **Placebo group** |
| 253.2167 | 8.59 | cis-9-palmitoleic acid | M-H[1-] | 1.24 (-0.51, 3.03) | -5.81 (-8.70, -2.83) |
| 265.2167 | 7.36 | 9-heptadecynoic acid | M-H[1-] | -0.26 (-2.44, 1.96) | 3.40 (0.49, 6.40) |
| 269.2115 | 7.19 | 3-keto palmitic acid | M-H[1-] | 0.05 (-0.52, 0.63) | -1.22 (-2.29, -0.13) |
| 279.2325 | 8.70 | 14Z,17-octadecadienoic acid | M-H[1-] | 0.73 (-0.24, 1.71) | -2.44 (-4.20, -0.64) |
| 340.2855 | 8.92 | Ceramide(d18:1/2:0) | M-H[1-] | 0.24 (-0.44, 0.92) | -1.32 (-2.56, -0.06) |
| 365.2348 | 8.70 | Tetrahydrocortisol | M-H[1-] | 0.16 (-0.37, 0.70) | -0.98 (-1.73, -0.21) |
| 370.7464 | 8.70 | PS(P-16:0/18:2(9Z,12Z)) | M-2H[2-] | 1.59 (-0.26, 3.48) | -3.97 (-6.84, -1.01) |
| 380.1937 | 8.70 | PA(14:1(9Z)/0:0) | M-H[1-] | 0.52 (-0.72, 1.77) | -2.48 (-4.53, -0.39) |
| 395.2202 | 8.50 | PA(15:0/0:0) | M-H[1-] | 1.90 (0.03, 3.81) | -3.37 (-6.02, -0.65) |
| 403.2682 | 8.50 | PI(P-18:0/15:0) | M-2H[2-] | 0.53 (0.07, 1.01) | -0.30 (-0.97, 0.37) |
| 433.2358 | 7.43 | PA(18:2(9Z,12Z)/0:0) | M-H[1-] | 0.27 (-0.56, 1.10) | 2.59 (1.18, 4.01) |
| 445.1901 | 5.02 | Estrone 3-glucuronide | M-H[1-] | 0.17 (-1.82, 2.20) | -4.82 (-7.56, -2.00) |
| 460.2588 | 7.43 | PA(20:3(8Z,11Z,14Z)/0:0) | M-H[1-] | 0.49 (-0.97, 1.97) | 3.95 (1.67, 6.29) |
| 465.2490 | 5.82 | Androsterone 3-glucuronide | M-H[1-] | 0.25 (-1.67, 2.21) | -3.06 (-5.09, -0.98) |
| 478.2935 | 7.83 | PE(18:1(9Z)/0:0) | M-H[1-] | -0.79 (-2.86, 1.33) | 4.00 (0.13, 8.03) |
| 504.3092 | 7.43 | PC(17:2(9Z,12Z)/0:0) | M-H[1-] | 0.36 (-0.98, 1.72) | 3.89 (1.80, 6.02) |
| 506.3270 | 8.39 | PC(17:1(9Z)/0:0) | M-H[1-] | 0.43 (-1.02, 1.89) | 3.86 (0.84, 6.97) |
| 528.3094 | 7.41 | PE(22:4(7Z,10Z,13Z,16Z)/0:0) | M-H[1-] | 0.01 (-1.48, 1.50) | 2.28 (0.65, 3.94) |
| 568.3418 | 7.33 | LysoPC(22:5(7Z,10Z,13Z,16Z,19Z)) | M-H[1-] | -0.77 (-2.28, 0.76) | 2.80 (0.23, 5.45) |
| 572.2970 | 7.43 | PS(22:4(7Z,10Z,13Z,16Z)/0:0) | M-H[1-] | -0.05 (-0.94, 0.84) | 2.17 (0.88, 3.47) |
| 595.2887 | 11.57 | PI(18:2(9Z,12Z)/0:0) | M-H[1-] | 0.44 (-0.57, 1.46) | 2.85 (1.49, 4.23) |
| 643.5275 | 11.62 | DG(20:2(11Z,14Z)/18:2(9Z,12Z)/0:0) | M-H[1-] | 0.41 (-0.90, 1.75) | -1.95 (-3.81, -0.04) |
| 720.4979 | 11.56 | PE(P-16:0/20:5(5Z,8Z,11Z,14Z,17Z)) | M-H[1-] | -1.01 (-3.58, 1.64) | 3.89 (0.28, 7.63) |
| 738.5101 | 11.56 | PC(18:4(6Z,9Z,12Z,15Z)/15:0) | M-H[1-] | -1.77 (-3.41, -0.11) | 2.93 (0.82, 5.09) |
| 746.5121 | 11.56 | PE(O-16:1(1Z)/22:6(4Z,7Z,10Z,13Z,16Z,19Z)) | M-H[1-] | -0.30 (-1.68, 1.10) | 2.41 (0.62, 4.23) |
| 759.6433 | 11.57 | SM(d18:0/20:0) | M-H[1-] | 0.78 (-0.06, 1.64) | -1.58 (-2.73, -0.43) |
| 765.5785 | 11.57 | PA(O-20:0/22:4(7Z,10Z,13Z,16Z)) | M-H[1-] | -2.25 (-4.94, 0.53) | 3.89 (0.35, 7.55) |
| 767.5469 | 11.57 | PC(20:4(5Z,8Z,11Z,14Z)/15:0) | M-H[1-] | -0.04 (-0.61, 0.53) | 0.93 (0.17, 1.68) |
| 816.4866 | 7.43 | PS(17:2(9Z,12Z)/22:6(4Z,7Z,10Z,13Z,16Z,19Z)) | M-H[1-] | -0.26 (-0.99, 0.48) | 1.79 (0.67, 2.93) |
| 845.5195 | 7.32 | PI(17:1(9Z)/18:2(9Z,12Z)) | M-H[1-] | -0.02 (-1.02, 0.99) | 2.36 (0.67, 4.08) |
| 883.5383 | 8.70 | PI(18:1(9Z)/20:4(5Z,8Z,11Z,14Z)) | M-H[1-] | -0.22 (-1.29, 0.85) | 1.72 (0.26, 3.21) |

**Abbreviation:** CIs, confidence intervals; PS, phosphatidyl serine; PA, phosphatidic acid; PI, phosphatidyl inositol; PE, phosphatidyl ethanolamine; PC, phosphatidyl choline; LysoPC, lysophosphatidyl choline; DG, Diacylglycerol; SM, sphingomyelin


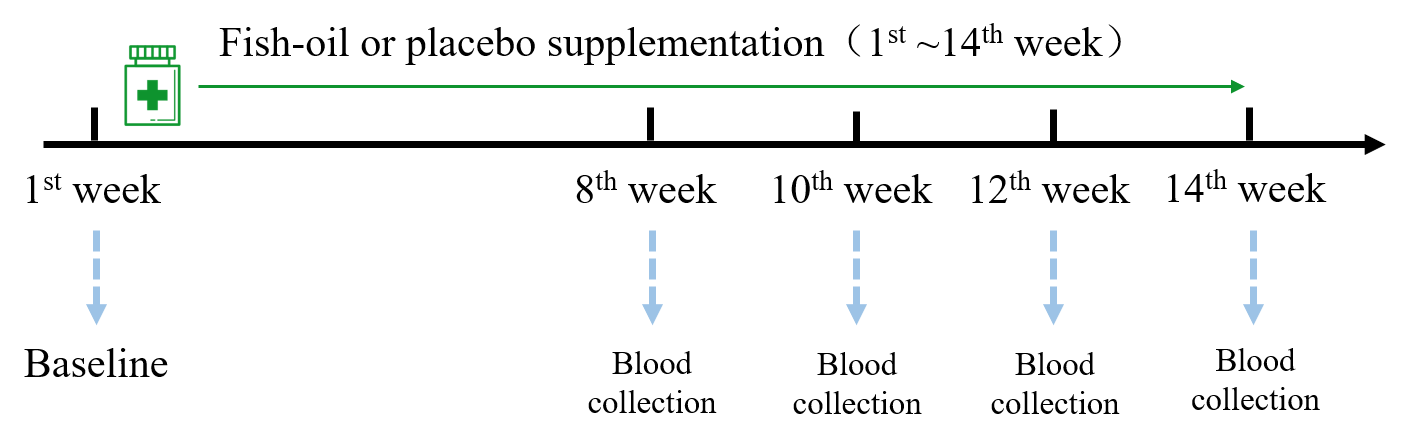
**Figure S1.** Timeline for the health measurements.

***Note.*** Placebo group was assigned to receive sunflower-seed oil intervention.


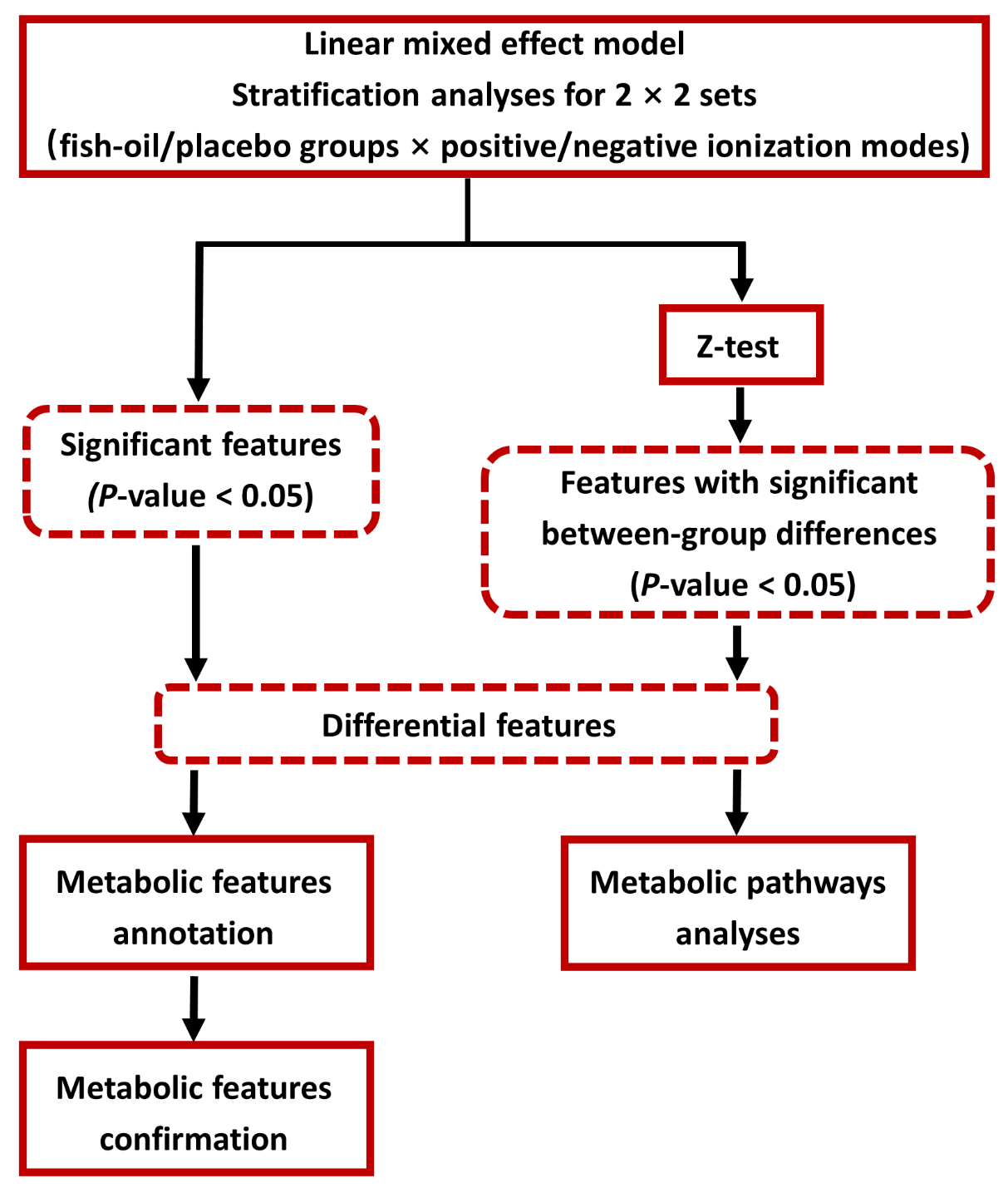
**Figure S2.** Flowchart of Metabolome-Wide Association Study analyses.

**
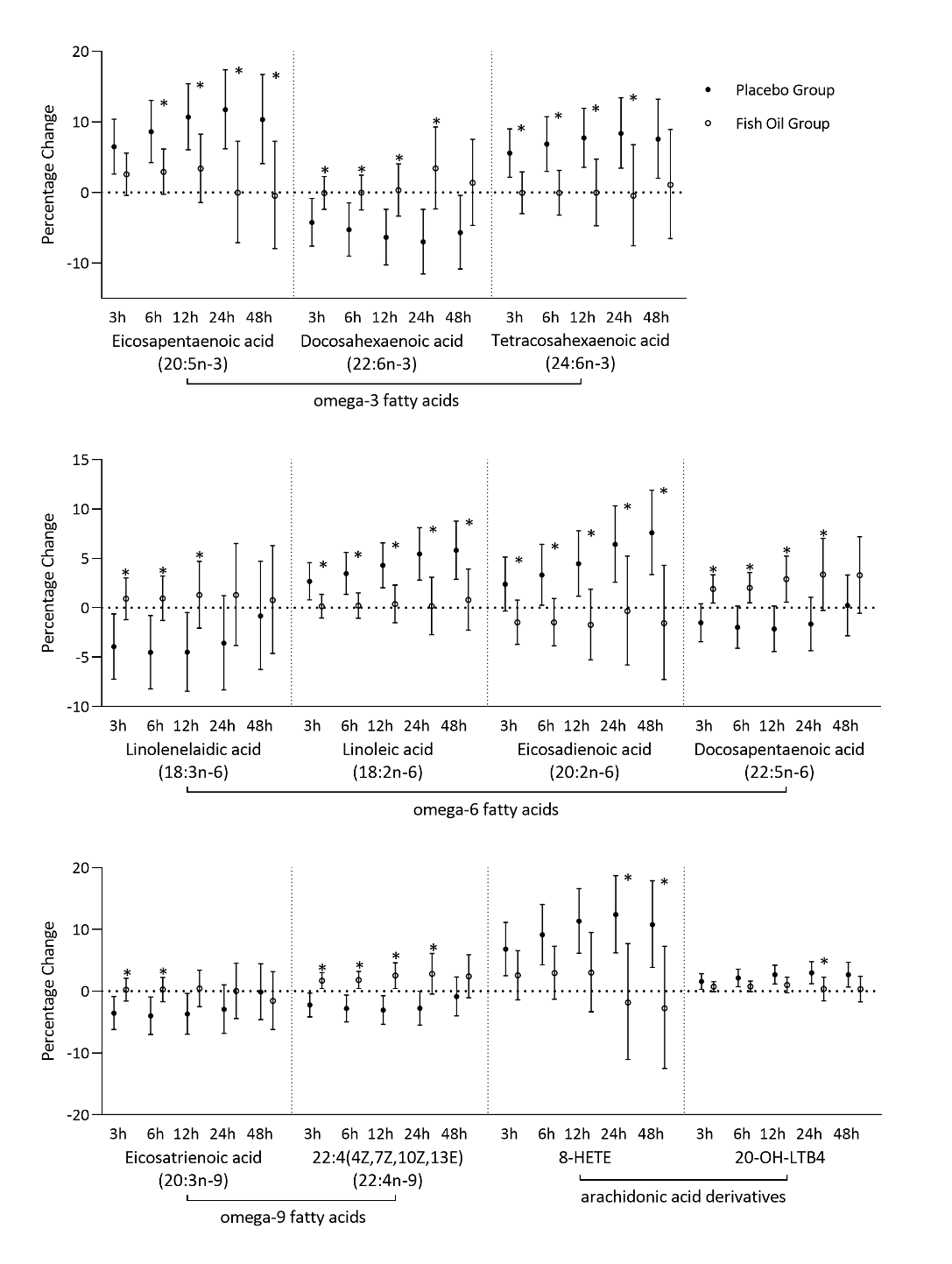
Figure S3.** Percentage changes in omega-3 fatty acids, omega-6 fatty acids, omega-9 fatty acids, and arachidonic acid derivatives associated with a 10-μg/m^3^ increment in ambient fine particulate matter (PM_2.5_) concentration after adjusting for baseline dietary intakes of nutrients in the sunflower-seed oil (placebo) group and the fish oil group.

**Abbreviations:** 8-HETE, 8-hydroxyeicosatetraenoic acid; 20-OH-LTB4, 20-OH-Leukotriene B4.

***Note.* *** Significant difference between groups (*P*-value < 0.05).

**
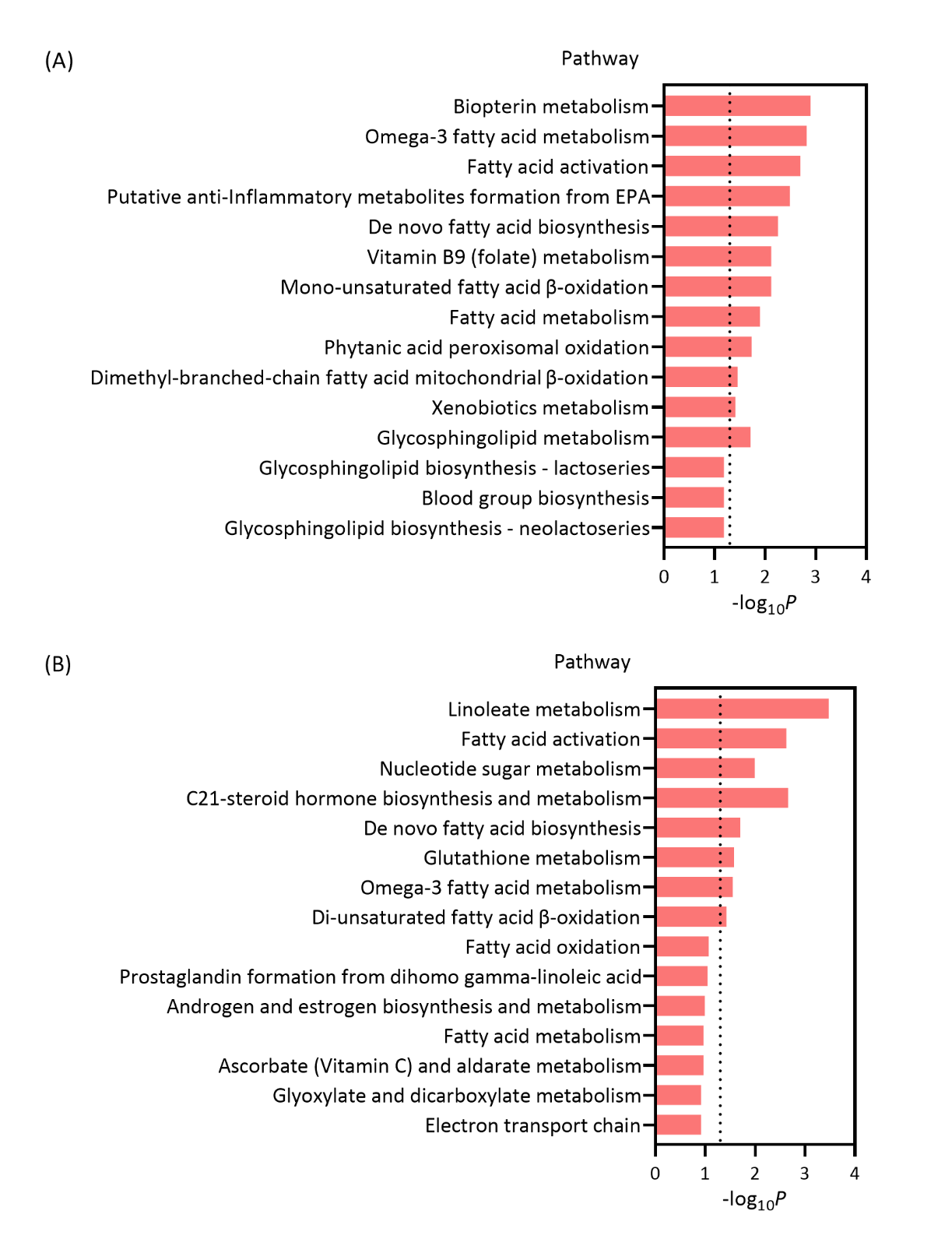
Figure S4.** Top 15 most enriched metabolic pathways after adjusting for baseline dietary intakes of nutrients in positive ionization mode (A), and negative ionization mode (B) at a lag of 0–6 hours.

**
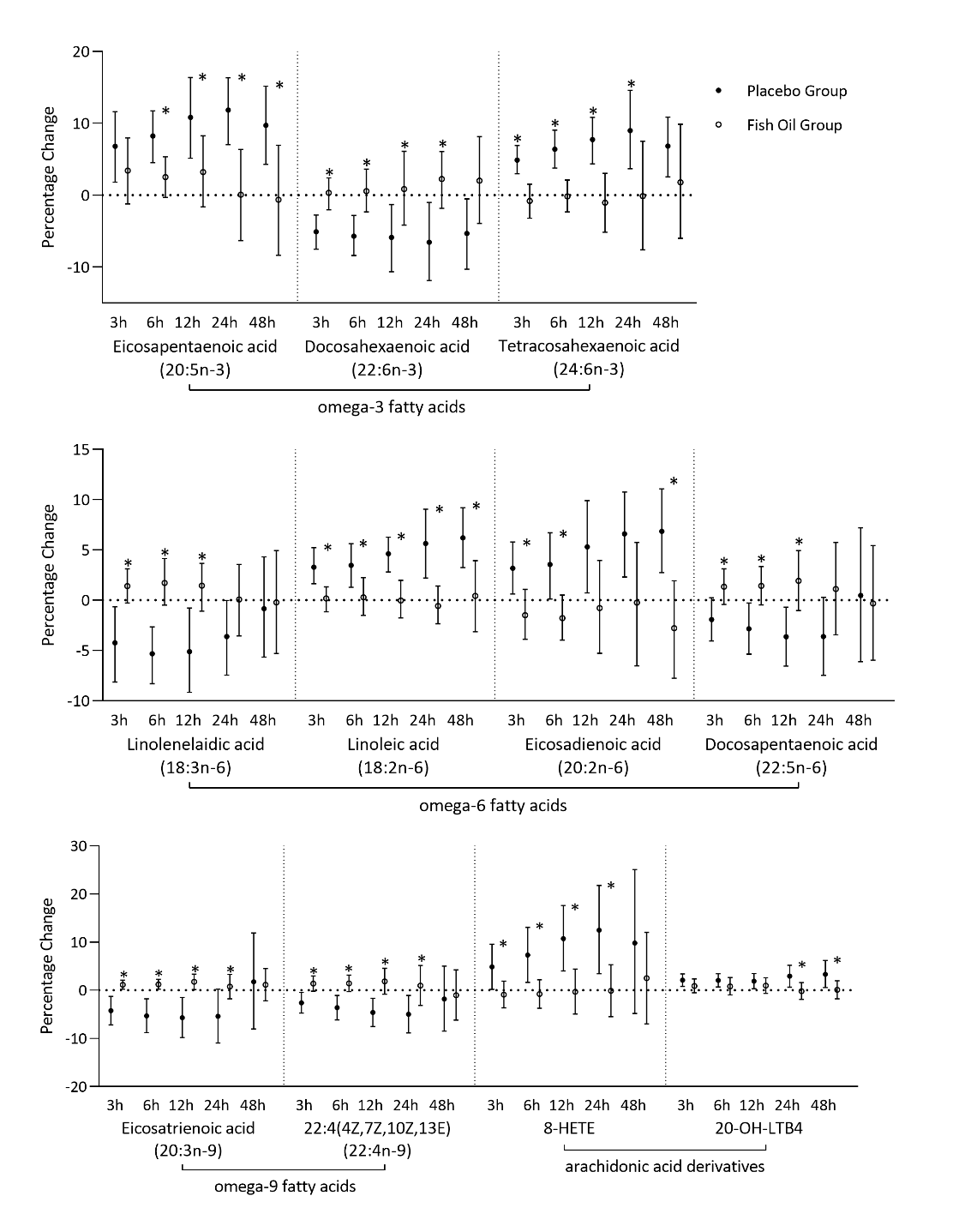
Figure S5.** Percentage changes in omega-3 fatty acids, omega-6 fatty acids, omega-9 fatty acids, and arachidonic acid derivatives associated with a 10-μg/m^3^ increment in ambient fine particulate matter (PM_2.5_) concentration after adjusting for concentration of nitrogen dioxide at lag 0–24 h in the sunflower-seed oil (placebo) group and the fish oil group.

Abbreviations as in Figure S3.

***Note.* *** Significant difference between groups (*P*-value < 0.05).

**
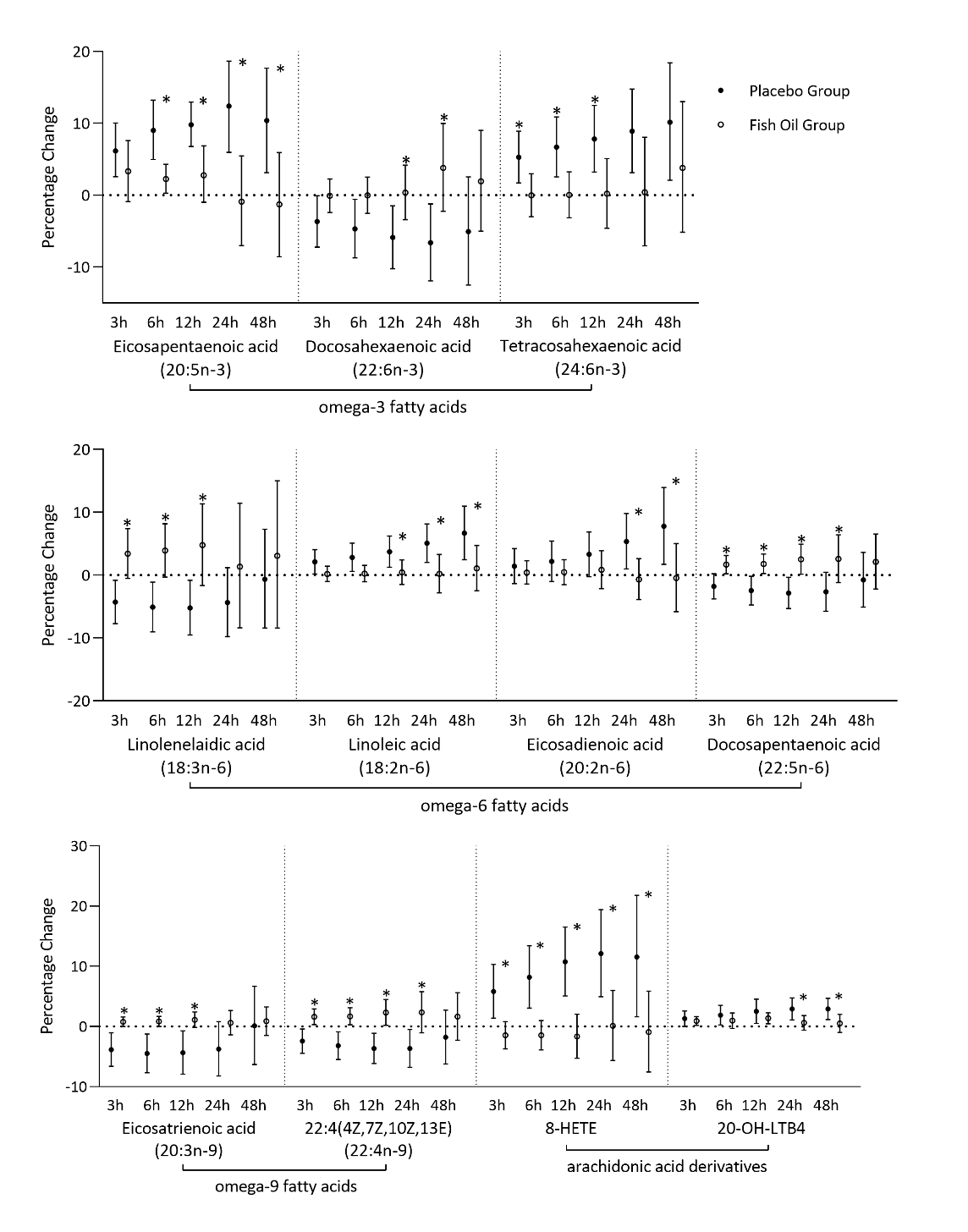
Figure S6.** Percentage changes in omega-3 fatty acids, omega-6 fatty acids, omega-9 fatty acids, and arachidonic acid derivatives associated with a 10-μg/m^3^ increment in ambient fine particulate matter (PM_2.5_) concentration after adjusting for concentration of sulfur dioxide at lag 0–24 h in the sunflower-seed oil (placebo) group and the fish oil group.

Abbreviations as in Figure S3.

***Note.* *** Significant difference between groups (*P*-value < 0.05).

**
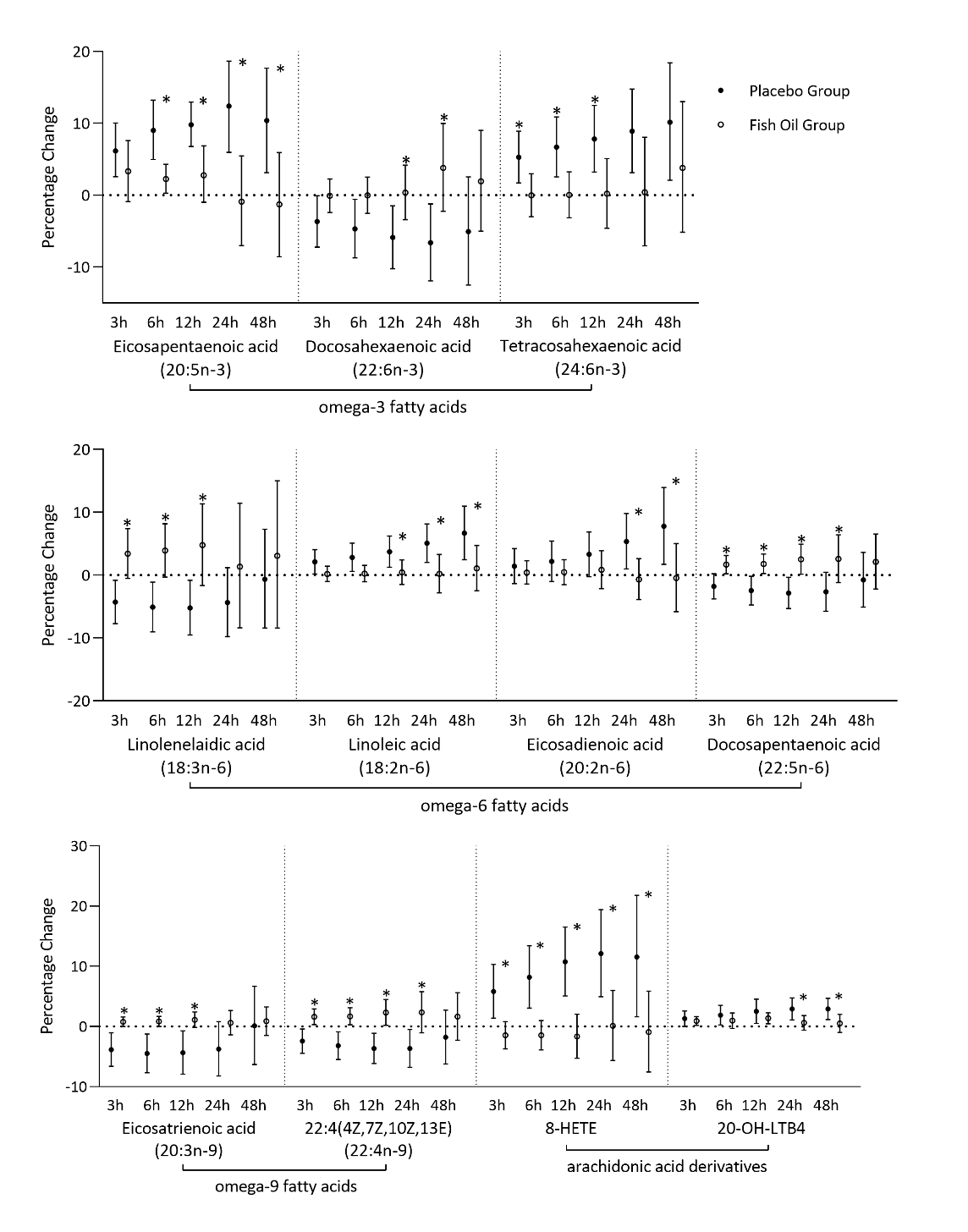
Figure S7.** Percentage changes in omega-3 fatty acids, omega-6 fatty acids, omega-9 fatty acids, and arachidonic acid derivatives associated with a 10-μg/m^3^ increment in ambient fine particulate matter (PM_2.5_) concentration after adjusting for concentration of ozone at lag 0–24 h in the sunflower-seed oil (placebo) group and the fish oil group.

Abbreviations as in Figure S3.

***Note.* *** Significant difference between groups (*P*-value < 0.05).

**
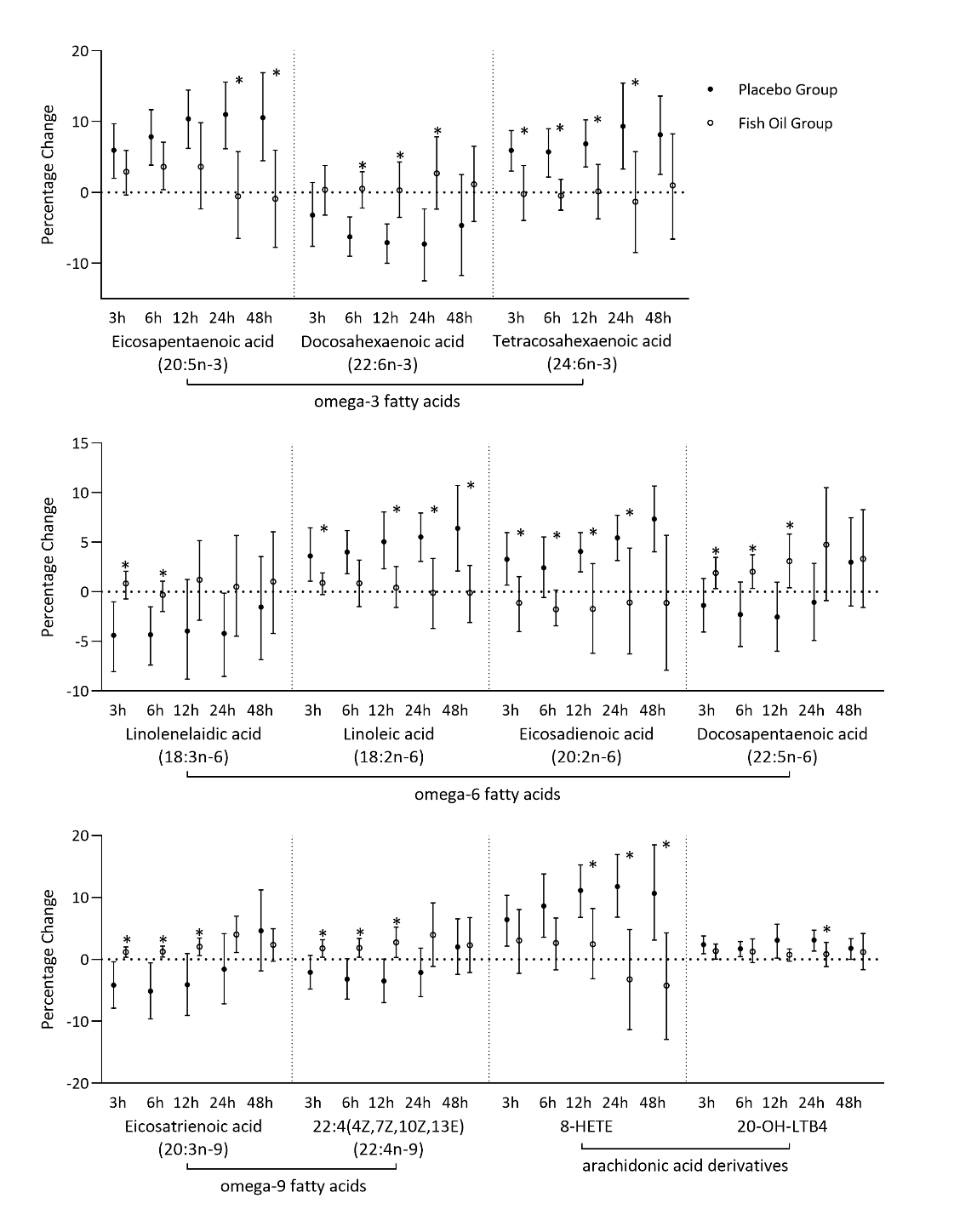
Figure S8.** Percentage changes in omega-3 fatty acids, omega-6 fatty acids, omega-9 fatty acids, and arachidonic acid derivatives associated with a 10-μg/m^3^ increment in ambient fine particulate matter (PM_2.5_) concentration after adjusting for concentration of carbon monoxide at lag 0–24 h in the sunflower-seed oil (placebo) group and the fish oil group.

Abbreviations as in Figure S3.

***Note.* *** Significant difference between groups (*P*-value < 0.05).
